# Reliability, validity and responsiveness of the German self-reported foot and ankle score (SEFAS) in patients with foot or ankle surgery

**DOI:** 10.1186/s12891-017-1772-1

**Published:** 2017-10-10

**Authors:** Dariusch Arbab, Katharina Kuhlmann, Christoph Schnurr, Bertil Bouillon, Christian Lüring, Dietmar König

**Affiliations:** 1 Department of Orthopedic Surgery, Klinikum Dortmund, Member Faculty of Health Witten/Herdecke University, Beurhausstraße 40, 44137 Dortmund, Germany; 2 Medical University Düsseldorf, Moorenstraße 5, 40225 Düsseldorf, Germany; 3 Department of Orthopedic Surgery, St. Vinzenz Krankenhaus, Schlossstraße 85, 40447 Düsseldorf, Germany; 4 Department of Traumatology and Orthopedic Surgery, Cologne-Merheim Medical Center (CMMC), University of Witten/ Herdecke, Ostmerheimer Str. 200, 51109 Cologne, Germany; 5 Department of Orthopedic Surgery, Klinikum Dortmund, Beurhausstraße 40, 44137 Dortmund, Germany; 6 LVR Clinics of Orthopedic Surgery, Horionstraße 2, 41479 Viersen, Germany

**Keywords:** SEFAS, Patient reported outcome measures, Questionnaire, Translation, Validation

## Abstract

**Background:**

Patient-reported outcome measures are a critical tool in evaluating the efficacy of orthopedic procedures and are increasingly used in clinical trials to assess outcomes of health care. The intention of this study was to develop and culturally adapt a German version of the Self-reported Foot and Ankle Score (SEFAS) and to evaluate reliability, validity and responsiveness.

**Methods:**

According to Cross Cultural Adaptation of Self-Reported Measure guidelines forward and backward translation has been performed. The German SEFAS was investigated in 177 consecutive patients. 177 Patients completed the German SEFAS, Foot and Ankle Outcome Score (FAOS), Short-Form 36 and numeric scales for pain and disability (NRS) before and 118 patients 6 months after foot or ankle surgery. Test-Retest reliability, internal consistency, floor and ceiling effects, construct validity and minimal important change were analyzed.

**Results:**

The German SEFAS demonstrated excellent test-retest reliability with ICC values of 0.97. Cronbach’s alpha (α) value of 0.89 demonstrated strong internal consistency. No floor or ceiling effects were observed for the German version of the SEFAS. As hypothesized SEFAS correlated strongly with FAOS and SF-36 domains. It showed moderate (ES/SRM > 0.5) responsiveness between preoperative assessment and postoperative follow-up.

**Conclusion:**

The German version of the SEFAS demonstrated good psychometric properties. It proofed to be a valid and reliable instrument for use in foot and ankle patients.

**Trial registration:**

DRKS00007585.

**Electronic supplementary material:**

The online version of this article (10.1186/s12891-017-1772-1) contains supplementary material, which is available to authorized users.

## Background

Patient reported outcome measurements (PROMS) can provide reliable and valid measures of patient’s degree of pain, impairment, disability, and quality of life. They are a critical tool in evaluating the efficacy of orthopaedic procedures and are increasingly used in clinical trials to assess outcomes of health care [1, 2]. The Self-reported Foot and Ankle Score (SEFAS) is a PROM for surgery of the foot and ankle, initially assessed in patients undergoing total ankle replacement due to osteoarthritis or inflammatory arthritis [3]. Further evaluation provided evidence in support of the reliability and validity using data from a large sample of patients undergoing surgery of a wide range of forefoot, hindfoot and ankle disorders [4]. In a systematic literature review about PROM’s in hallux valgus the SEFAS showed good psychometric properties with good availability and less items compared to the Manchester-Oxford Foot Questionnaire [5]. The SEFAS is based on the New Zealand total ankle questionnaire [6], and was adapted by Cöster et al. in 2012 [3]. It contains 12 items, with 5 response options. The questionnaire covers different constructs, which are not reported separately in subscales. Pain, limitation of function and other symptoms are the main constructs [3]. Currently it has not been translated and evaluated in other languages.

The aim of this study was to translate the SEFAS into German language, culturally adapt it according to international guidelines, and to evaluate reliability, validity and responsiveness.

## Methods

The study was approved by Ethics Commission of the Faculty of Medicine of Cologne University (ref 15–252, DRKS-ID DRKS00007585, IRB00003528 Klinikum der Universitat zu Koln IRB #1, 2014–04-28) and performed in accordance with the Declaration of Helsinki. Written informed consent from all participants was obtained.

### Translation

Forward and backward translation of the SEFAS was performed according to international guidelines of the International Society for Pharmacoeconomics and Outcomes Research (ISPOR) [7]. Two bilingual translators with different profiles (one physician) whose native language was German independently translated the English version forward into German. Two native English speakers without medical education performed backward translation of the provisional German questionnaire into English. Translators and a group of foot and ankle surgeons consolidated all versions of the questionnaire and developed a final version for field testing. The final version was tested on 15 patients with foot or ankle disorders to ascertain acceptance and comprehension (Additional files 1 and 2).

### Patients and validation procedure

From November 2014 to January 2016 a total of 177 patients undergoing surgery of the foot or ankle were consecutively recruited at a single institution. Eligibility criteria included adult patients undergoing primary foot or/and ankle surgery for osteoarthritis, deformity, rheumatoid arthritis, impingement of the ankle, tendon disorders or bone defect. Patients were asked to complete the German SEFAS, the German Foot and Ankle Outcome Score (FAOS) the German Short-Form 36 Health Survey (SF-36) and a numeric scale for pain and disability (NRS). SEFAS, FAOS, SF-36 and NRS were completed 3–14 days before surgery (t1) and again on the morning before surgery (t2) for reliability testing. 6 months after surgery (t3) all participants were asked to complete SEFAS a last time.

### Instruments

The SEFAS is a 12-item questionnaire covering different constructs, which are not separately reported in subscales. We defined 3 subscales consisting of: pain (p) (seven items, question 1, 2, 5, 8, 9, 11, 12), (limitation of) function (lof) (three items question 3, 6, 7) and others (o) (2 items question 4, 10). Patients score each question on a five-point Likert scale scored from 0 to 4, with 0 representing the worst stage and the sum of 48 representing normal function [3].

The FAOS is a 42-item instrument to evaluate symptoms and functional limitations related to the foot and ankle [8]. It consists of five domains: pain (p), other symptoms (s), activities of daily living (adl), sport and recreational activities (s/r), and foot-and ankle-related quality of life (qol). Each item is scored on a five-point Likert scale from 0 to 4, with 4 representing the worst stage. The total score is transformed to a scale from 0 to 100, like SEFAS low numbers represent more severe stages. FAOS has been translated into German and validated as a reliable instrument for use in foot and ankle patients [9].

The SF-36 instrument is a widely used generic patient-reported instrument to measure health related quality of life. It consists of eight domains: physical functioning (pf), role physical (rp), role emotional (re), social functioning (sf), mental health (mh), energy/vitality (e/v), pain (p), general health perception (gh). It has been translated and validated into German [10].

The NRS were used to determine pain and disability of the foot and ankle. On a 0–10 scale 10 represents the most severe pain or disability.

### Statistical analysis

The SEFAS and FAOS subscale scores were entered into a Microsoft Excel spreadsheet (Microsoft Corporation, Redmond WA) and analyzed using SPSS v24 (SPSS Inc. Chicago, Illinois). A *p*-value <0.05 was considered to indicate statistical significance.

### Reliability

#### Reproducibility

Reproducibility as test-retest reliability was assessed by calculating interclass correlation coefficient (ICC, Two-way Random Effect Model Absolute Agreement Definition) between SEFAS completed at the first visit 3–14 days before surgery (step 1) and second time before surgery (step 2). An ICC value of 0.7 and above was considered as good [11, 12].

#### Internal consistency

Reliability also includes internal consistency [12]. Internal consistency is the extent to which items within a scale are homogeneous, thus measuring the same construct [13, 14]. Cronbach’s alpha (α) coefficient was calculated to assess internal consistency of the SEFAS items. Values of alpha of 0.7, 0.8 and 0.9 are considered to represent fair, good and excellent degree of internal consistency, respectively [15].

#### Floor and ceiling effects

Floor and ceiling effects were considered to exist if more than 15% of responses reached lowest or highest possible score. A high floor or ceiling effect could make it difficult to measure changes after intervention like surgery [3, 16, 17].

### Validity

#### Construct validity

Describes the extent to which a score relates to other scores [17]. As no gold standard exists the SEFAS and its defined subscales were compared to FOAS and SF-36 and NRS pain and disability using non-parameteric correlation coefficients (Spearman’s Rho). Correlation coefficients <0.4 were considered as low, 0.4–0.59 as moderate and 0.6–0.79 as high correlation [18]. For convergent validity Cöster et al. 2014 expected high correlation between SEFAS and the FAOS domains p, adl, qol and with the SF-36 domains pf and bp [4]. We hypothesized for the SEFAS p a high correlation with FAOS domain p, adl and SF-36 domain bp, pf and NRS. SEFAS lof and the FAOS dimension sr,adl, qol and SF-36 domain pf should show high correlations as well. For discriminant validity low to moderate correlations were expected between SEFAS and SF-36 domain gh, re and mh [4].

#### Responsiveness

Responsiveness is the extent to which a questionnaire is able to detect changes over time or due to an intervention such as surgery [19]. All patients completed SEFAS before surgery (t1) and 6 months after surgery (t3). To test responsiveness effect size (ES) and standardized response means (SRM) were calculated. ES is calculated as the difference between the means before and after intervention divided by the standard deviation (SD) of the same measure before treatment [12]. SRM is calculated as the difference between the means before and after treatment divided by the SD of the change. For both, ES and SRM, values of 0.2, 0.5 and 0.8 were regarded as small, moderate and large effects, respectively [12].

#### Minimal important change (MIC) (laut Dawson ist hier “minimal important change” der richtige Ausdruck – Siehe meine letzte mail)

Minimal important change (MIC) is the smallest change in a treatment outcome that a patient or physician would identify as important. MIC describes a threshold above which outcome is experienced as relevant by the patient and avoids the problem of bare statistical significance [20]. One distribution-based approach to calculate MIC is the minimal detectable change (MDC). It is defined as minimum amount of change that can be considered above the threshold of a measurement error. If the change in a score is higher than MDC, it can be considered as a true change [21]. It is calculated from the standard error of measurement (SEM), which is related to the internal consistency/reliability of the score (Cronbach’s alpha). (SEM = Standard deviation *√1- Cronbach’s alpha). To allow comparisons with other studies, the MDC was calculated based on the confidence level of 90% (MDC90: MDC = 1,65 * SEM * √2) [22]. In order to estimate significant change of the scores over time, we performed likelihood ratio tests after applying mixed-effects linear regression with random patient effects to account for repeated (longitudinal) measurements on the same patient [23, 24].

## Results

One hundred seventy seven patients, 130 women and 47 men, with a mean age of 57 years (18–92) undergoing surgery of the foot or ankle were consecutively recruited at a single institution and completed the baseline 3–14 days before surgery (t1). On the morning before surgery (t2) 145 patients completed MOXFQ to determine reliability. 6 months after surgery (t3) 117 patients completed MOXFQ, FAOS, SF-36 and NRS to test responsiveness. 118 patients were undergoing forefoot, 56 patients hindfoot or ankle surgery.

### Reliability

#### Reproducibility

The SEFAS demonstrated excellent test-retest reliability with ICC values of 0.95 for limitation of function, 0.97 for pain, 0.96 other symptoms and 0.97 for the SEFAS total. The mean indexes for the baseline and the reliability assessments were 48.4 (Standard deviation (SD) 19.7) and 49.7 (SD 20.7), respectively (Table 1).

**Table 1:** Reliability - Cronbach’s alpha and ICC

		Reliabilität				Consistency
		Test Mean t1 (SD)	Retest Mean t2 (SD)	Cronbach’s α t1	Cronbach’s α t2	ICC
SEFAS	pain	52.05 (19.29)	52.69 (19.33)	0.858	0.870	0.973 (0.960;0.981)
	lof	47.13 (23.93)	48.54 (24.90)	0.747	0.790	0.954 (0.934;0.968)
	os	45.92 (27.57)	47.49 (29.48)	0.223	0.354	0.959 (0.941;0.972)
	total	48.37 (19.65)	49.68 (20.68)	0.881	0.889	0.970 (0.957;0.979)

Reliability with test-retest reliability and internal consistency of the German SEFAS

lof = limitation of function, o = others, ICC = interclass correlation coefficient, SD = standard deviation, t1 = first assessment, t2 = second assessment, CI = confidence interval

#### Internal consistency

Cronbach’s alpha (α) value of 0.89 for SEFAS total and 0.87 for pain demonstrated strong internal consistency. Lof values of 0.79 still showed fair internal consistency, whereas os demonstrated low consistency (0.35) (Table 1).

#### Floor and ceiling effects

No floor or ceiling effects were observed for the German version of the SEFAS (Table 2).

**Table 2:** Floor and ceiling effects

SEFAS	t1				t2			
	pain	lof	os	total	pain	lof	os	total
min (0)	0.7%	4.1%	9.0%	0.7%	0.7%	4.1%	8.2%	0%
max (100)	0%	0%	3.5%	0%	0%	0%	4.8%	0%
all (min + max)	0.7%	4.1%	12.5%	0.7%	0.7%	4.1%	13%	0%

Floor and ceiling effects are considered to be present when more than 15% of the individuals reach the highest or lowest possible numeric value of a score

t1 = 3–14 days before surgery, t2 = morning before surgery, lof = limitation of function, os = others

### Validity

#### Construct validity

To examine construct validity the Spearman’s correlation coefficients between SEFAS, SF-36, FAOS and NRS were examined and shown in Table 3. We hypothesized that SEFAS subscale os will show low correlation to any of the FAOS domains. Convergent validity of the SEFAS was shown with strong correlations (0.6–0.79) with the FAOS and its subscales and SF-36 domains pf, gph and bp. As hypothesized SEFAS p subscale correlated strongly with FAOS domain p, adl, and SF-36 domains bp and pf. SEFAS lof and FAOS sr, adl, qol and SF-36 domain pf showed high correlation as expected. Moderate correlation could be calculated for SEFAS and NRS, whereas SEFAS p and NRS showed high construct validity. Discriminant validity with low to moderate correlation could be calculated for SF-36 gh, re, mh and gmh. All these findings were statistically significant (*p* < 0.05).

**Table 3:** Validity

*N* = 177		SEFAS			
		pain	limitation of function	other symptoms	total
		Spearman	Spearman	Spearman	Spearman
FAOS	symptoms	0.595	0.565	0.441	0.625
	pain	0.792	0.748	0.444	0.765
	activities of daily living	0.792	0.778	0.418	0.771
	sports/recreation	0.739	0.725	0.371	0.712
	quality of life	0.706	0.672	0.293	0.642
	total	0.801	0.766	0.434	0.775
SF36	physical functioning	0.691	0.665	0.391	0.673
	role physical	0.631	0.578	0.355	0.597
	bodily pain	0.777	0.691	0.306	0.673
	general health	0.470	0.398	0.272	0.433
	Energy/vitality	0.377	0.373	0.145	0.332
	social functioning	0.638	0.513	0.270	0.528
	role emotional	0.470	0.477	0.298	0.479
	mental health	0.379	0.347	0.099	0.301
	general physical health	0.718	0.683	0.403	0.685
	general mental health	0.416	0.389	0.181	0.358
NRS	total	0.617	0.508	0.283	0.542

Construct validity: Baseline data and assessment of Spearman’s correlation coefficients and of SEFAS, FAOS, SF-36 and NRS

#### Responsiveness

Table 4 shows the responsiveness of the SEFAS. With an effect size of 0.71 the SEFAS demonstrated moderate (ES/SRM > 0.5) responsiveness between preoperative assessment (t2) and postoperative follow-up (t3) indicating that a good degree of change was detected following surgery. The highest effect size showed the pain subscale (1.06).

**Table 4:** Responsiveness

		Responsiveness					MID	
		Per-OP Mean (SD)	Post-OP Mean (SD)	Mean Difference (SD)	ES	SRM	MDC 90	SEM
SEFAS	pain	52.06 (19.25)	29.37 (23.47)	22.69 (22.82)	1.06	0.99	4.97	213
	lof	47.51 (25.30)	29.20 (25.39)	18.31 (25.55)	0.72	0.72	5.58	2.39
	os	51.14 (27.59)	47.39 (27.78)	3.75 (32.32)	0.14	0.12	7.03	3.01
	total	50.37 (20.58)	35.33 (21.83)	15.04 (23.18)	0.71	0.65	5.04	2.16

Responsiveness of the SEFAS showing mean changes, effect size and minimal important difference demonstrating minimal detectable change and standard error of measurement

lof = limitation of function, o = others, SD = standard deviation, ES = effect size, SRM = standardized response means, MID = minimal important difference, MDC = minimal detectable change, SEM = standard error of measurement

The SEM was 2.39, 2.13, 3.01 and 2.16 for the German SEFAS lof, p, os and total, respectively. MDC90 (90% confidence level) were 5.58, 4.97, 7.03 and 5.04 for domains lof, p, os and total, respectively. The mean difference (15.04) between preoperative and postoperative assessment is shown on Table 4. The German SEFAS showed higher changes than the MDC (5.04) which indicates true changes [19].

## Discussion

Limited internationally used self-reported instruments are available to assess outcome of foot and ankle surgery. There is a need for reliable, valid, free and in native languages available reported outcome measures used for clinical trials and health care evaluation. The SEFAS has been initially validated for patients with ankle osteoarthritis and later for patients with a wide variety of different disorders of the foot and ankle proofing good validity, reliability and responsiveness [3, 4]. It is available in English and Swedish and has not been culturally adapted and translated in any other language.

In this study the English version of the SEFAS was cross-culturally adapted and translated to German according to the official guidelines of the International Society for Pharmacoeconomics and Outcomes Research (ISPOR) [7]. Originally the questionnaire covers different constructs, which are not reported separately in subscales 3]. We defined the main constructs pain, limitation of function and others as subscales to enhance comparability to FAOS and SF-36 domains.

The psychometric analyses demonstrated that the German version of the SEFAS has good validity, reliability and responsiveness in patients undergoing foot and ankle surgery.

Test-retest reliability of the German SEFAS showed excellent results with ICC values ranging from 0.97 for the SEFAS and 0.95 for limitation of function, 0.97 for pain and 0.96 other symptoms. These results are comparable to the Swedish version [4]. The mean indexes for the baseline and the reliability assessments were 48.4 (Standard deviation (SD) 19.7) and 49.7 (SD 120.7), respectively.

Strong internal consistency has been demonstrated for the SEFAS (Cronbach’s alpha 0.89) and SEFAS pain (0.87) of the German version. The limitation of function (0.79) subscale showed fair internal consistency, whereas the other symptoms subscale demonstrated low consistency (0.35). Low internal consistency for os subscale was expected due to inhomogeneous questions asking for different constructs. The Swedish SEFAS showed similar results (0.84) for internal consistency. No floor or ceiling effects were observed, which is in line with the Swedish version.

Construct validity was determined by comparing the German SEFAS with the German SF-36 and FAOS. The German SEFAS showed strong correlations (>0,6) with FAOS and its subscales and SF-36 domains physical functioning, general physical health and bodily pain. As hypothesized SEFAS pain subscale correlated strongly with FAOS domains pain and activity of daily living, SF-36 domains bodily pain and physical functioning. SEFAS domain limitation of functioning showed strong correlation with FAOS sports/recreation, activities of daily living and quality of life and with SF-36 domain physical functioning. Cöster et al. 2014 could confirm 80% of their predefined hypotheses showing strong correlations between SEFAS and 4 of the 5 subscales of FAOS [4]. In our study the German SEFAS showed strong correlations with all FAOS subscales. Discriminant validity with low to moderate correlation was found for the German SEFAS and SF-36 domains general health, role emotional, mental health and general physical health.

Table 4 illustrates the responsiveness of the German SEFAS which demonstrated moderate (ES/SRM > 0.5) responsiveness in 118 patients with an effect size of 0.71 between preoperative (t2) and postoperative follow-up (t3). Cöster et al. 2014 showed higher effect sizes (ES >1) in their population (70 patients) [4].

Evaluation of the German SEFAS showed similar results to the Swedish SEFAS [4]. Cultural differences, smaller number of patients and different foot pathologies and surgeries may be the reason for differing results in some aspects. A definition of subscales was not intended by the developers of the questionnaire but allowed a more specific examination of psychometric properties.

A limitation of the study is the inhomogeneity of the patient sample regarding sex and age. We see this as a result of the included pathologies which concern mainly women and are not restricted in age of occurrence. For this reason, these heterogeneities can also be found in comparable studies.

Unfortunately it was not possible to evaluate reliability and responsiveness based on the total sample size of 177 patients due to a lower response rate in repeated assessments. However, a sample size of >100 patients could be achieved at all time which we consider sufficient and representative. Regarding the assessment of Minimal important change, in our study only distribution-based methods were used. These are not as patient-centered as anchor-based methods and do not include a clinical criterion which limits their interpretability in a clinical setting.

## Conclusions

In conclusion the German translation of the SEFAS demonstrated good psychometric properties. Our study demonstrated that the German questionnaire is a valid and reliable instrument for patients with foot and ankle disorders and can be used as a tool for evaluating the efficacy of surgical procedures and in clinical trials to assess outcomes of health care.

## Additional files

Additional file 1: German Translation of SEFAS Questionnaire. (PDF 317 kb)

Additional file 2: English SEFAS Questionnaire. (PDF 32 kb)

## Abbreviations

adl: subdomain activities of daily living
e/v: Subdomain energy/vitality
ES: Effect size (ES)
FAOS: Foot and Ankle Outcome Score
gh: Subdomain general health perception
ICC: Interclass correlation coefficient
MDC: minimal detectable change
mh: Subdomain mental health
MIC: Minimal important change
NRS: Numeric scales for pain and disability
p: Subdomain pain
p: Subdomain pain (p)
pf: Subdomain physical functioning
Qol: Subdomain quality of life (qol)
re: Subdomain role emotional
rp: Subdomain role physical
s: Subdomain other symptoms
s/r: Subdomain sport and recreational activities
SEFAS: Self-reported Foot and Ankle Score
SEM: standard error of measurement
sf: Subdomain
SF36: Short-Form 36
SRM: Standardized response means
