# Supplementary material for: Reliability, validity and responsiveness of the German self-reported foot and ankle score (SEFAS) in patients with foot or ankle surgery
Source: BMC Musculoskelet Disord. 2017 Oct 10;18:409. doi: 10.1186/s12891-017-1772-1 (PMC5634858; doi:10.1186/s12891-017-1772-1)
Supplement: Supplementary file 1 — German Translation of SEFAS Questionnaire. (PDF 317 kb) [file 12891_2017_1772_MOESM1_ESM.pdf]

„SEFAS": Fragebogen zu Fuß- und Sprunggelenk

Deutsche Version

Datum: _____/______/______ Geburtsdatum: _____/______/______

Name: ______________________________________

Wir möchten Sie bitten die folgenden 12 Fragen zu beantworten. Jede Frage hat 5 Antwortmöglichkeiten, die von 0-4 entsprechend ihres Schweregrades eingeteilt sind.
4 = geringster Schweregrad und 0 = höchster Schweregrad

Bitte kreuzen Sie die Antwort an, die Ihren Zustand während der **letzten 4 Wochen** am besten beschreibt.

1. Wie würden Sie die Schmerzen beschreiben, die Sie gewöhnlich im betroffenen Fuß/Sprunggelenk empfinden?
4 ☐ keine
3 ☐ sehr geringe
2 ☐ geringe
1 ☐ mäßige
0 ☐ starke

2. Wie lange konnten Sie gehen, bis starke Schmerzen im betroffenen Fuß/Sprunggelenk aufgetreten sind?
4 ☐ mehr als 30 min ohne Schmerzen
3 ☐16-30 min
2 ☐ 5-10 min
1 ☐ kurze Strecken in und um das Haus
0 ☐ aufgrund starker Schmerzen nicht möglich

3. War es Ihnen möglich, auf unebenem Boden zu gehen?
4 ☐ ja, ohne Schwierigkeiten
3 ☐ mit geringen Schwierigkeiten
2 ☐ mit mäßigen Schwierigkeiten
1 ☐ mit starken Schwierigkeiten
0 ☐ nein, nicht möglich

4. Mussten Sie eine orthopädische Einlage, eine Fersenerhöhung oder spezielle Schuhe verwenden?
4 ☐ nie
3 ☐ gelegentlich
2 ☐ oft
1 ☐ meistens
0 ☐ immer

5. Wie stark haben Sie die Schmerzen im betroffenen Fuß/Sprunggelenk bei Ihrer üblichen Arbeit, Hausarbeit und Hobbies, beeinträchtigt?
4 ☐ gar nicht
3 ☐ ein wenig
2 ☐ mäßig
1 ☐ stark
0 ☐ vollständig

6. Hatten Sie aufgrund Ihres betroffenen Fußes/Sprunggelenks einen hinkenden Gang?
4 ☐ an keinem Tag
3 ☐ an ein bis zwei Tagen
2 ☐ an einigen Tagen
1 ☐ an den meisten Tagen
0 ☐ jeden Tag

7. Konnten Sie Treppen steigen?
4 ☐ ja, ohne Schwierigkeiten
3 ☐ mir geringen Schwierigkeiten
2 ☐ mit mäßigen Schwierigkeiten
1 ☐ mit starken Schwierigkeiten
0 ☐ nicht möglich

8. Haben Sie die Schmerzen im betroffenen Fuß/Sprunggelenk nachts im Bett beeinträchtigt?
4 ☐ an keiner Nacht
3 ☐ an ein bis zwei Nächten
2 ☐ an einigen Nächten
1 ☐ an den meisten Nächten
0 ☐ jede Nacht

9. Wie stark haben die Schmerzen im betroffenen Fuß/Sprunggelenk Ihre Freizeitaktivitäten beeinträchtigt?
4 ☐ gar nicht
3 ☐ ein wenig
2 ☐ mäßig
1 ☐ stark
0 ☐ vollständig

10. War Ihr Fuß geschwollen?
4 ☐ gar nicht
3 ☐ gelegentlich
2 ☐ oft
1 ☐ meistens
0 ☐ immer

11. Wie stark waren Ihre Schmerzen im betroffenen Fuß/Sprunggelenk, wenn Sie von einem Stuhl aufgestanden sind (z.B. nach dem Essen)?
4 ☐ keine Schmerzen
3 ☐ geringe Schmerzen
2 ☐ mäßige Schmerzen
1 ☐ starke Schmerzen
0 ☐ unerträgliche Schmerzen

12. Hatten Sie im betroffenen Fuß/Sprunggelenk plötzlich einschießende Schmerzen, ein Stechen oder Krämpfe?
4 ☐ an keinem Tag
3 ☐ an ein bis zwei Tagen
2 ☐ an einigen Tagen
1 ☐ an den meisten Tagen
0 ☐ jeden Tag

Vielen Dank, dass Sie diese Fragen beantwortet haben
