# Supplementary material for: Reliability, validity and responsiveness of the German self-reported foot and ankle score (SEFAS) in patients with foot or ankle surgery
Source: BMC Musculoskelet Disord. 2017 Oct 10;18:409. doi: 10.1186/s12891-017-1772-1 (PMC5634858; doi:10.1186/s12891-017-1772-1)
Supplement: Supplementary file 2 — English SEFAS Questionnaire. (PDF 32 kb) [file 12891_2017_1772_MOESM2_ESM.pdf]

Date when answering: __ __ — __ __ — __ __ __ __
D D M M Y Y Y Y

SELF-REPORTED FOOT & ANKLE QUESTIONNAIRE (SEFAS)

We would like you to answer the 12 questions below. Each question is graded from 0- 4
4 = the mildest or least troublesome and 0 = the most severe or most troublesome.

Please cross the box that best describes your condition during the last 4 weeks

1. How would you describe the pain you usually have from the foot/ankle in question?<br><br>4 ☐ None<br>3 ☐ Very mild<br>2 ☐ Mild<br>1 ☐ Moderate<br>0 ☐ Severe	5. How much has the pain from the foot/ankle in question interfered with your usual work including housework and hobbies?<br><br>4 ☐ Not at all<br>3 ☐ A bit<br>2 ☐ Moderately<br>1 ☐ Greatly<br>0 ☐ Totally
2. For how long have you been able to walk before severe pain arises from the foot/ ankle in question?<br><br>4 ☐ No pain up 30 min.<br>3 ☐ 16-30 minutes<br>2 ☐ 5-10 minutes<br>1 ☐ Around the house only<br>0 ☐ Unable_to walk at all because of severe pain	6. Have you been limping when walking because of the foot/ankle in question?<br>4 ☐ No days<br>3 ☐ Only one or two days<br>2 ☐ *Some days*<br>1 ☐ Most days<br>0 ☐ Every day
3. Have you been able to walk on uneven ground?<br><br>4 ☐ Yes, easily<br>3 ☐ With little_difficulty<br>2 ☐ With moderate difficulty<br>1 ☐ With extreme_difficulty<br>0 ☐ No impossible	7. Have you been able to climb *a flight of* stairs?<br><br>4 ☐ Yes, easily<br>3 ☐ With little_difficulty<br>2 ☐ With moderate difficulty<br>1 ☐ With extreme trouble<br>0 ☐ Impossible
4. Have you had to use an orthotic (shoe insert), heel lift or special shoes?<br><br>4 ☐ Never<br>3 ☐ Occasionally<br>2 ☐ Often<br>1 ☐ Most of the time<br>0 ☐ Always	8. Have you been troubled by pain from the foot/ ankle in question in bed at night?)<br><br>4 ☐ No night)<br>3 ☐ Only one or two nights<br>2 ☐ Some nights<br>1 ☐ Most nights<br>0 ☐ Every night
9. How much has pain from the foot/ankle in question affected your usual recreational activities?<br><br>4 ☐ Not at all<br>3 ☐ A bit<br>2 ☐ Moderately<br>1 ☐ Greatly<br>0 ☐ Totally	11. After a meal (sat at a table) how painful has it been for you to stand up from a chair because of the foot/ankle in question?<br><br>4 ☐ Not at all painful<br>3 ☐ Slightly painful<br>2 ☐ Moderately painful<br>1 ☐ Very painful<br>0 ☐ Unbearable
10. Have you had swelling of your foot?<br><br>4 ☐ None at all<br>3 ☐ Occasionally<br>2 ☐ Often<br>1 ☐ Most of the time<br>0 ☐ All the time	12. Have you had a severe sudden pain shooting, stabbing or spasms from the foot/ankle in question?<br><br>4 ☐ No days<br>3 ☐ Only one or two days<br>2 ☐ Some day<br>1 ☐ Most days<br>0 ☐ Every day

THANK YOUR FOR COMPLETING THESE QUESTIONS
